# Combining the quantitative faecal immunochemical test and full blood count reliably rules out colorectal cancer in a symptomatic patient referral pathway

**DOI:** 10.1007/s00384-021-04079-2

**Published:** 2021-12-21

**Authors:** Mark S. Johnstone, Paul Burton, Georgios Kourounis, Jack Winter, Emilia Crighton, David Mansouri, Paul Witherspoon, Karen Smith, Stephen T. McSorley

**Affiliations:** 1 Academic Unit of Surgery, School of Medicine, University of Glasgow, Glasgow, UK; 2 eHealth, Corporate Services, Business Intelligence, NHS Greater Glasgow and Clyde, Glasgow, UK; 3 Department of Gastroenterology, Glasgow Royal Infirmary, NHS Greater Glasgow and Clyde, Glasgow, UK; 4 Public Health, Health Service, Public Health Screening, NHS Greater Glasgow and Clyde, Glasgow, UK; 5 Department of Coloproctology, Glasgow Royal Infirmary, NHS Greater Glasgow and Clyde, Glasgow, UK; 6 Department of Colorectal Surgery, Queen Elizabeth University Hospital, NHS Greater Glasgow and Clyde, Glasgow, UK; 7 Department of Clinical Biochemistry, Glasgow Royal Infirmary, NHS Greater Glasgow and Clyde, Glasgow, UK

**Keywords:** FIT, Faecal, Immunochemical, Test, Symptomatic, Colorectal

## Abstract

**Purpose:**

Faecal Immunochemical Test (FIT) has proven utility for Colorectal Cancer (CRC) detection in symptomatic patients. Most studies have examined FIT in symptomatic patients subsequently referred from primary care. We investigated associations between CRC and FIT in both referred and non-referred symptomatic patients.

**Methods:**

A retrospective, observational study of all patients with a FIT submitted Aug 2018 to Jan 2019 in NHS GG&C was performed. Referral to colorectal/gastroenterology and decision to perform colonoscopy were recorded. FIT results were grouped as f-Hb < 10/10–149/150–399/ ≥ 400 μg/g. The MCN cancer registry identified new cases of CRC. Covariables were compared using the *χ*^2^ test. Multivariate binary logistic regression identified independent predictors of CRC.

**Results:**

A total of 4968 patients were included. Raised FIT correlated with decision to refer (*p* < 0.001) and scope (*p* < 0.001). With 23-month median follow-up, 61 patients were diagnosed with CRC. These patients were older (median 69 vs 59 years, cancer and no cancer respectively, *p* = 0.001), more likely to be male (55.7% vs 42.1%, *p* = 0.033), and to report rectal bleeding (51.7% vs 36.1%, *p* = 0.013). FIT (< 10 µg/g 8.2% vs 76.7% and ≥ 400 µg/g 55.7% vs 3.8%, *p* < 0.001) and anaemia (45.9% vs 19.7%, *p* < 0.001) were associated with CRC. On multivariate analysis, age (*p* = 0.023), male sex (*p* = 0.04), FIT (≥ 400 OR 54.256 (95% CI:20.683–142.325; *p* < 0.001)), and anaemia (OR 1.956 (1.071–3.574; *p* = 0.029)) independently predicted CRC. One patient (0.04%) with a negative FIT and normal haemoglobin had CRC.

**Conclusion:**

GP referral and secondary care investigation patterns were influenced by FIT. The combination of normal Hb and f-Hb excluded CRC in 99.96% of cases, providing excellent reassurance to those prioritising access to endoscopy services.

**Supplementary Information:**

The online version contains supplementary material available at 10.1007/s00384-021-04079-2.

## Introduction

Colorectal cancer is the 4th most common cancer in the UK, with approximately 42,000 new cases and 16,500 deaths each year [1]. The NICE NG12 [2] and NHS Scotland Suspected Cancer Guidelines [3] have similar recommendations in terms of high risk lower gastrointestinal symptoms which should trigger an urgent suspicion of cancer referral. These include rectal bleeding with no obvious cause, persistent change in bowel habit (> 4 weeks, particularly diarrhoea), palpable abdominal or rectal masses, abdominal pain with weight loss, and unexplained iron deficiency anaemia, each of which may be tempered with the patient’s age [2, 3]. However, lower GI symptoms are poor predictors of colorectal cancer, with similar symptoms seen in both significant bowel disease (colorectal cancer, high risk adenoma, or inflammatory bowel disease) and functional bowel disorders [4]. Indeed, following introduction of the above referral guidance, while the number of referrals increased significantly, the proportion of patients found to have a colorectal cancer decreased and there was no change in cancer staging at diagnosis [5, 6]. The NICE DG30 guideline recommends that the faecal immunochemical test (FIT) be used to guide referral for suspected colorectal cancer in patients with lower risk lower GI symptoms (those without rectal bleeding but other unexplained symptoms that do not meet urgent suspected cancer pathway criteria) [7]. A number of health boards across the UK have introduced FIT submission as part of their colorectal urgent suspected cancer pathway [3]. Emerging data has proven the utility of FIT in symptomatic patients with sensitivity and specificity reportedly ranging from 85 to 100% and 56 to 91% respectively for colorectal cancer detection at a threshold of ≥ 10 µg Hb/g faeces [8–15]. FIT has proven equally valid in determining cancer risk in patients meeting high risk (NG12) or low risk (DG30) symptom criteria [6, 8, 16], and in patients with and without rectal bleeding [17]. Most studies to date have examined such FIT programmes and only included referred patients. However, few studies have assessed the real-life impact of the introduction of FIT on GP’s referral practice and colorectal surgeon’s and gastroenterologist’s decision to investigate further with colonoscopy. We aimed to examine associations between colorectal cancer diagnosis, symptoms, faecal haemoglobin concentration, and anaemia in patients both referred and not referred from primary care following the introduction of FIT into a symptomatic lower GI referral pathway.

## Methods

A retrospective, observational study was conducted to include all patients with a FIT submitted from primary care between August 2018 and January 2019 in NHS Greater Glasgow and Clyde (the period during which FIT was introduced to local referral pathways). FIT specimen collection devices (Minaris Medical America, Inc) with accompanying pictorial instructions and return envelopes were supplied to all GP practices as an adjunct to guide referral for patients with lower GI symptoms. Patients were asked to collect a single faecal sample and return to their GP practice as soon as possible. The kits were transported at ambient temperature via routine specimen collection services and stored at 4 °C prior to analysis in a single centralised laboratory. Analysis was carried out Monday to Friday so that most samples were analysed on day of receipt. Results were reported electronically to the requesting GP. Only FIT samples submitted from primary care from patients aged ≥ 16 years old were included.

To identify study participants, a search of the clinical biochemistry repository was conducted to capture all FIT samples submitted between August 2018 and January 2019. These samples were then interrogated, and where duplicate entries were identified, the first valid sample was kept. To obtain patient demographics and outcomes, cross-referencing of the SCI Store, SCI Gateway, Unisoft, CRIS, and MCN Cancer Registry were performed with the CHI number used as the linkage variable. A search of SCI store (Scottish Care Information Store Version 8.5) allowed the identification of patient demographics and blood results. Post codes were used to determine each patient’s Scottish Index of Multiple Deprivation (SIMD) score. SIMD is a measure of an area’s deprivation according to income, employment, education, health, access to services, crime, and housing [18]. SCI Gateway (Scottish Care Information Gateway R 20.0) was searched to identify referral letters from primary care to general surgery or gastroenterology within 3 months prior or after FIT collection. These letters were manually screened to identify lower GI symptoms and coded as rectal bleeding, persistent diarrhoea, other change in bowel habit, weight loss, abdominal pain, anal pain, faecal soiling, rectal mass, and abdominal mass. Referral letters were also used to identify patient co-morbidity. For the purposes of analysis, asthma and COPD were grouped as “respiratory disease”; ischaemic heart disease, cerebrovascular disease, peripheral vascular disease, and hypertension were grouped as “cardiovascular disease”; and previous diagnosis of Crohn’s, ulcerative colitis, or indeterminate colitis were groups as “inflammatory bowel disease.” Unisoft (Unisoft Medical Systems GI Reporting Tool) was used to identify all patients who underwent a colonoscopy following their FIT collection date. CRIS (Central Data Networks Radiology Information System) identified all patients who had a CT colonography, CT chest abdomen, and pelvis or CT abdomen and pelvis as their only form of investigation following referral. The MCN cancer registry was searched to identify all new diagnoses of colorectal cancer up to November 2020. Caldicott guardian approval was given by NHS GG&C to safeguard the record linkage with ethical approval waived for the purposes of service development.

Patients were categorised into 3 groups: “Not Referred” (FIT sample submitted from primary care but no accompanying referral letter to general surgery or gastroenterology), “Referred but not Scoped” (FIT sample submitted with accompanying referral but no colonoscopy), and “Referred and Scoped” (FIT sample submitted with accompanying referral and colonoscopy performed). Importantly, patients were only regarded as “referred” if a referral was made from primary care to general surgery or gastroenterology as part of the outpatient symptomatic lower GI referral pathway under investigation. Referrals out with this pathway to alternative specialities or emergency attendances were not regarded as “referred.” FIT results were grouped by f-Hb concentrations of < 10 μg/g, 10–149 µg/g, 150–399 µg/g, and ≥ 400 µg/g. Patients were defined as anaemic (male < 130 mg/L, female < 120 mg/L) based on WHO guidelines [19] and iron deficient (ferritin < 15 µg/L) based on BSG guidelines [20].

Covariables were compared using crosstabulation and the *χ*^2^ test. A value of *p* < 0.05 was considered statistically significant. To identify covariables which independently predicted colorectal cancer risk, univariate followed by multivariate binary logistic regression was performed. Selected covariables found to have a significant impact on colorectal cancer risk from the *χ*2 analysis were carried into the regression analysis. This allowed calculation of odds ratios (ORs) and 95% confidence intervals (95% CI). Covariables significant on univariate analysis (*p* < 0.05) were entered into a multivariate model using the backwards conditional method in which variables with a significance of *p* < 0.1 were removed from the model in a stepwise fashion. Statistical analysis was performed using SPSS software (SPSS Inc., Chicago, Illinois, USA).

## Results

### Referral pathway

**Fig. 1:**
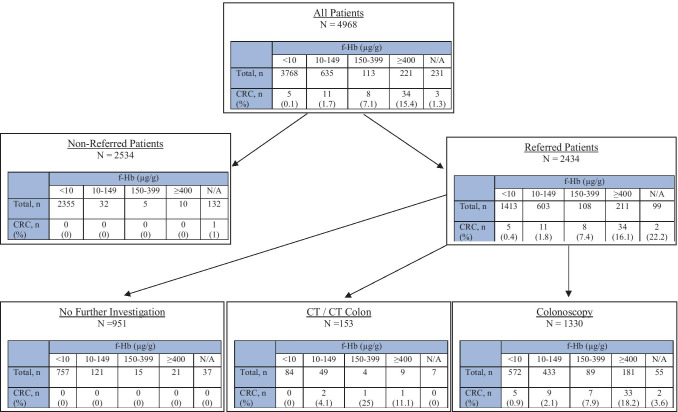
Investigation and referral pathway

The investigation and referral pathway of all 4968 adult patients with a FIT sample submitted from primary care between August 2018 and January 2019 in NHS GG&C can be seen in Fig. 1. Median age was 59 years (range 16 to 97). 2102 (42.3%) patients were male, and 2866 (57.7%) were female. A total of 3768 (75.8%) had f-Hb < 10 µg/g and 969 (19.5%) f-Hb ≥ 10 µg/g, with 635 (12.8%) between 10 and 149 µg/g, 113 (2.3%) between 150 and 399 µg/g, and 221 (4.4%) ≥ 400 µg/g. A total of 231 (4.6%) samples could not be processed by the laboratory and were not repeated.

Of the 4968 total, 2434 patients (49.0%) were subsequently referred to general surgery or gastroenterology. 2194 (90.1%) referral letters indicated the presence of any red flag symptom, with 887 (36.4%) patients reporting rectal bleeding, 602 (24.7%) persistent diarrhoea, 1207 (49.6%) other change in bowel habit, 466 (19.1%) weight loss, 796 (32.7%) abdominal pain, 77 (3.2%) anal pain, 150 (6.2%) faecal soiling, 44 (1.8%) rectal mass, and 60 (2.5%) abdominal mass. Of the 2434 referred patients, 1330 (54.6%) went on to have a colonoscopy. Of the 1104 referred patients who did not undergo colonoscopy, 153 (13.9%) had radiological imaging as their only modality of investigation: 63 (41.2%) CT colonography, 28 (18.3%) CT abdomen and pelvis, and 62 (40.5%) CT chest abdomen and pelvis.

A comparison between referred and not referred patients and between those patients who were or were not selected for colonoscopy can be seen in Supplementary Tables 1 and 2 respectively. Notably, patients who were referred were significantly older (median 60 vs 57 years, *p* < 0.001), had significantly higher f-Hb (≥ 10 μg/g 37.9% vs. 1.9%, *p* < 0.001), were more likely to be anaemic (22.4% vs 17.6%, *p* < 0.001), and had a greater proportion of iron deficiency anaemia (6.8% vs 3.9%, *p* < 0.001). Patients selected for colonoscopy were significantly younger (median 60 vs 61 years, *p* = 0.02), more likely to have reported PR bleeding (44.1% vs 27.3%, *p* < 0.001) or persistent diarrhoea (27.4% vs 21.5%, *p* = 0.001), had significantly higher f-Hb (≥ 10 μg/g 52.9% vs 19.8%, *p* < 0.001), and more iron deficiency anaemia (8.0% vs 5.4%, *p* = 0.018).

A total of 47 patients had a raised f-Hb (32 between 10 and 149 µg/g, 5 between 150 and 399 µg/g, and 10 ≥ 400 µg/g) but were not referred to the general surgery or gastroenterology service. The records of each of these patients were reviewed. Ten patients were deemed too frail for referral by their GP following positive FIT. Seven patients had a recent acute inpatient admission and investigation, or decision not to investigate had been organised from that admission. Ten patients were already known to general surgery or gastroenterology and were regularly seen on an outpatient basis including patients scheduled for surveillance colonoscopy. Three patients were referred to care of the elderly rather than general surgery or gastroenterology. Seventeen patients had a positive FIT but no clear reason for no onward referral.

### Colorectal cancer cases

With a median 23-month (range 21–26) follow-up, 61 patients (1.2%) were diagnosed with a colorectal cancer. Of these, 56 belonged to the Referred and Scoped group with the diagnosis confirmed at colonoscopy as a direct result of referral. Four patients in the Referred but Not Scoped group were diagnosed with a colorectal malignancy. Of these, two were deemed too frail for colonoscopy and following referral underwent CT abdomen and pelvis which identified a colorectal cancer for which both had supportive management only. One patient underwent a CT colonography following referral and proceeded straight to laparoscopic right hemicolectomy with tissue diagnosis only confirmed postoperatively. One patient was referred from primary care but prior to clinic review presented with small bowel obstruction secondary to a caecal cancer and underwent an emergency right hemicolectomy. Finally, one patient belonged to the Not Referred group. This patient’s submitted FIT could not be processed by the laboratory and was not repeated. The patient was later admitted as an emergency with symptomatic anaemia and had a cancer diagnosed at inpatient colonoscopy.

Table 1 compares those diagnosed with a cancer and those who were not. Patients diagnosed with a cancer were significantly older (median age 69 vs 59 years, *p* = 0.001), more likely to be male (55.7% vs 42.1%, *p* = 0.033), have a history of inflammatory bowel disease (IBD) (2.1% vs 0.3%, *p* = 0.04), have reported rectal bleeding (51.7% vs 36.1%, *p* = 0.013), and significantly less likely to have reported abdominal pain (20.0% vs 33.0%, *p* = 0.034). Faecal haemoglobin was significantly associated with colorectal malignancy (< 10 µg/g 8.2% vs 76.7% and FIT ≥ 400 µg/g 55.7% vs 3.8% cancer and no cancer respectively, *p* < 0.001), as did the presence of anaemia (45.9% vs 19.7%, *p* < 0.001), iron deficiency anaemia (ferritin < 15) (26.2% vs 5.1%, *p* < 0.001), and both normocytic (26.2% vs 15.6%) and microcytic anaemia (18.0% vs 2.8%, *p* < 0.001).

**Table 1:** Comparison between all patients diagnosed with a cancer and those who were not

		Colorectal Cancer		*p*
		Yes	No	
*N*		61	4907	
Age	Median (range)	69 (36–95)	59 (16–97)	< 0.001
	< 50	7 (11.5%)	1448 (29.5%)	
	50–74	30 (49.2%)	2622 (53.4%)	
	≥ 75	24 (39.3%)	837 (17.1%)	
Sex	Male	34 (55.7%)	2068 (42.1%)	0.033
	Female	27 (44.3%)	2839 (57.9%)	
Scottish Index of Multiple Deprivation	1 (most deprived)	16 (26.2%)	1470 (30.0%)	0.59
	2	13 (21.3%)	855 (17.4%)	
	3	11 (18.0%)	610 (12.4%)	
	4	8 (13.1%)	758 (15.4%)	
	5 (least deprived)	13 (21.3%)	1214 (24.7%)	
Co-morbidity^a^	Respiratory disease	8 (17.0%)	270 (16.7%)	0.95
	Diabetes	6 (12.8%)	197 (12.2%)	0.902
	Cardiovascular disease	9 (19.1%)	250 (15.4%)	0.489
	IBD	1 (2.1%)	5 (0.3%)	0.04
Symptoms^b^	Rectal bleeding	31 (51.7%)	856 (36.1%)	0.013
	Persistent diarrhoea	16 (26.7%)	586 (24.7%)	0.725
	Other change in bowel habit	27 (45.0%)	1180 (49.7%)	0.472
	Weight loss	12 (20.0%)	454 (19.1%)	0.865
	Abdominal pain	12 (20.0%)	784 (33.0%)	0.034
	Anal pain	0 (0%)	77 (3.2%)	0.156
	Faecal soiling	4 (6.7%)	146 (6.1%)	0.869
	Rectal mass	1 (1.7%)	43 (1.8%)	0.934
	Abdominal mass	1 (1.7%)	59 (2.5%)	0.686
QFIT	< 10	5 (8.2%)	3763 (76.7%)	< 0.001
	10–149	11 (18.0%)	624 (12.7%)	
	150–399	8 (13.1%)	105 (2.1%)	
	≥ 400	34 (55.7%)	187 (3.8%)	
	N/A	3 (4.9%)	228 (4.6%)	
Anaemia^b^	No	33 (54.1%)	3351 (80.3%)	< 0.001
	Yes	28 (45.9%)	820 (19.7%)	
Iron deficiency anaemia (Ferritin < 15)^d^	No	45 (73.8%)	3905 (94.9%)	< 0.001
	Yes	16 (26.2%)	210 (5.1%)	
Anaemia and MCV^e^	Not anaemic	33 (54.1%)	3351 (80.3%)	< 0.001
	Macrocytic anaemia (MCV > 100)	1 (1.6%)	54 (1.3%)	
	Normocytic anaemia (MCV 80–100)	16 (26.2%)	649 (15.6%)	
	Microcytic anaemia (MCV < 80)	11 (18.0%)	117 (2.8%)	

^a^Data missing for 3302 (66.5%) patients

^b^Data missing for 2534 (15.0%) patients

^c^Data missing for 736 (14.8%) patients

^d^Data missing for 792 (15.9%) patients

^e^Data missing for 736 (14.8%) patients

At a f-Hb threshold of 10 μg/g, sensitivity for colorectal cancer was 91.80%, specificity 80.42%, negative predictive value (NPV) 99.87%, and positive predictive value (PPV) 5.47%. The number of colonoscopies (number needed to scope, NNS) that would have to be performed to diagnose one colorectal cancer at the 10 μg/g threshold was 18.

On multivariate analysis (Table 2), increasing age (50–74 years OR 2.749 (95% CI: 1.150–6.572; *p* = 0.023) and ≥ 75 years OR 4.140 (95% CI: 1.610–10.641; *p* = 0.003)), male sex (OR 1.817 (95% CI: 1.027–3.216; *p* = 0.04)), FIT (10–149 µg/g OR 4.623 (95% CI: 1.587–13.465; *p* = 0.005), 150–399 µg/g OR 19.690 (95% CI: 6.207–62.459; *p* < 0.001), and ≥ 400 µg/g OR 54.256 (95% CI: 20.683–142.325; *p* < 0.001)), and anaemia (OR 1.956 (1.071–3.574; *p* = 0.029)) retained significance as independent predictors of colorectal cancer.

**Table 2:** Univariate and multivariate binary logistic regression analysis of factors impacting on likelihood of colorectal cancer diagnosis

		Univariate			Multivariate		
		OR	95% C.I	*p*	OR	95% C.I	*p*
Age	< 50	1.0			1.0		
	50–74	2.367	1.037–5.402	0.041	2.749	1.150–6.572	0.023
	≥ 75	5.931	2.545–13.825	< 0.001	4.140	1.610–10.641	0.003
Sex	Female	1.0			1.0		
	Male	1.729	1.04–2.874	0.035	1.817	1.027–3.216	0.04
Rectal bleeding	No	1.0			1.0		
	Yes	1.896	1.135–3.167	0.015	1.004	0.535–1.883	0.990
f-Hb (μg/g)	< 10	1.0			1.0		
	10–149	13.267	4.594–38.313	< 0.001	4.623	1.587–13.465	0.005
	150–399	57.341	18.448–178.229	< 0.001	19.690	6.207–62.459	< 0.001
	≥ 400	136.836	52.911–353.882	< 0.001	54.256	20.683–142.325	< 0.001
Anaemia	No	1.0			1.0		
	Yes	3.467	2.084–5.770	< 0.001	1.956	1.071–3.574	0.029

### Combination of FIT and anaemia to rule out colorectal cancer

**Fig. 2:**
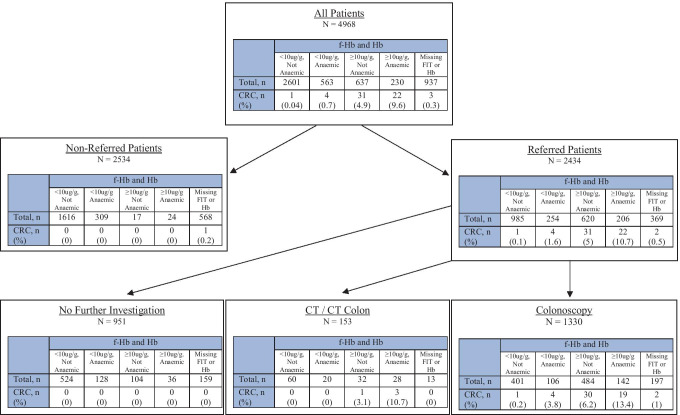
Investigation and referral pathway with combined FIT and anaemia measures

There was a significant association between a raised FIT and anaemia: 563 of 3164 (17.8%) patients with FIT < 10 µg/g were anaemic as compared to 136 of 561 (24.2%) of those with FIT 10–149 µg/g, 32 of 104 (30.8%) FIT 150–399 µg/g, and 62 of 202 (30.7%) FIT ≥ 400 µg/g (*p* < 0.001). However, despite this relationship, FIT and anaemia were both found to be independent predictors of colorectal cancer and were therefore next combined. A total of 4031 of 4968 (81.1%) patients in the study had both a valid FIT and haemoglobin. Combining FIT and Hb, 2601 patients had a negative FIT and were not anaemic, 563 had a negative FIT but were anaemic, 637 had a positive FIT but were not anaemic, and 230 had both a positive FIT and were anaemic. Table 3 shows a comparison between these four groups of patients, and Fig. 2 shows the investigation and referral pathway of all patients in the study with this combined FIT and anaemia measure. Four patients (0.7%) with a negative FIT but anaemia, 31 patients (4.9%) with a positive FIT but normal haemoglobin, and 22 patients (9.6%) with both a positive FIT and anaemia were diagnosed with a colorectal cancer. Only 1 patient (0.04%) with a negative FIT and normal haemoglobin was diagnosed with a colorectal cancer. Combining FIT at a f-Hb threshold of 10 μg/g with the presence or absence of anaemia resulted in a sensitivity for colorectal cancer of 98.28%, specificity 65.44%, NPV 99.96%, and PPV 3.99%. NNS is 26.

**Table 3:** Comparison by combined FIT and anaemia for all patients with both a valid FIT and full blood count

		f-Hb < 10 μg/g Not anaemic	f-Hb < 10 μg/g Anaemic	f-Hb ≥ 10 μg/g Not anaemic	f-Hb ≥ 10 μg/g Anaemic	*p*
N		2601	563	637	230	
Age	Median (range)	57 (16–93)	69 (23–94)	60 (17–97)	75 (19–97)	< 0.001
	< 50	847 (32.6%)	78 (13.9%)	176 (27.6%)	30 (13.0%)	
	50–74	1454 (55.9%)	297 (52.8%)	343 (53.8%)	85 (37.0%)	
	≥ 75	300 (11.5%)	188 (33.4%)	118 (18.5%)	115 (50.0%)	
Sex	Male	1072 (41.2%)	225 (40.0%)	291 (45.7%)	99 (43.0%)	0.155
	Female	1529 (58.8%)	338 (60.0%)	346 (54.3%)	131 (57.0%)	
Colorectal cancer		1 (0.04%)	4 (0.7%)	31 (4.9%)	22 (9.6%)	< 0.001

## Discussion

This study provides a comprehensive description of the use of FIT in symptomatic patients during its initial period of use in NHS GG&C. This is one of few studies to include all patients with a FIT submitted from primary care regardless of onwards referral or decision to perform colonoscopy, reflecting real-life practice. By using cancer registry data with long follow-up, we have been able to capture all cancer cases rather than only cancers diagnosed following referral and scope. The results suggest that FIT is actively influencing GPs in their decision of whether to refer to colorectal and gastroenterology services and is influencing hospital doctors in their decision to perform colonoscopy. Additionally, these results add to the evidence that whilst symptoms should act as a trigger for assessment with FIT, they are poor predictors of the presence of cancer. In keeping with prior studies, only the presence of rectal bleeding significantly correlates with malignancy [17]. However, rectal bleeding did not remain an independent predictor of colorectal cancer on multivariate analysis. In contrast, FIT and the presence of anaemia were both independent predictors of cancer. Combining a FIT at a f-Hb threshold of 10 μg/g with the absence of anaemia was able to effectively exclude colorectal cancer in 99.96% of cases, which should provide excellent reassurance to general practitioners and specialist practitioners. Patients with a f-Hb < 10 μg/g and without anaemia represented 64.5% of patients. With appropriate safety netting in place, these patients can be reassured.

There are a wide variety of sensitivities and specificities reported in the literature for colorectal cancer detection in symptomatic patients (85 to 100% and 56 to 91% respectively at ≥ 10 µg Hb /g faeces threshold) [8–15]. Several systematic review and meta-analyses have tried to amalgamate the available data. Westwood et al. [10] pooled data from 9 such studies. A total of 4091 patients were tested with the OC-Sensor FIT test and 507 patients with HM-JACKarc. At the 10 µg/g threshold, pooled sensitivity and specificity were 92.1% and 85.8% for OC-Sensor and 100% and 76.6% for HM-JACKarc. Pin Vieito et al. [11] conducted a systematic review and meta-analysis which included 13,073 patients from 15 different studies. There was significant heterogeneity in the studies with a wide range of f-Hb thresholds used and with both symptomatic and screening patients included. However, pooled estimates for sensitivity and specificity at the 10 µg/g threshold where only studies with solely symptomatic patients were included (*n* = 4035) were 94.1% and 66.0%.

Other studies have compared the use of FIT in patients with high or low risk symptoms as per the NICE NG12 criteria [2] and NICE DG30 criteria [7] respectively. The NICE FIT study [6] reported on 9822 patients referred to 50 English hospitals as urgent suspected colorectal cancer, who subsequently underwent colonoscopy. A total of 7194 (73.2%) patients had high risk symptoms as per the NICE NG12 criteria, 1994 (20.3%) patients had low risk symptoms meeting the NICE DG30 criteria, and 634 (6.5%) had other symptoms warranting urgent referral. At a FIT threshold of 10 µg/g, sensitivity and specificity for colorectal cancer for those with high-risk symptoms were 92.2% and 82.3% respectively. For those with low-risk symptoms, sensitivity was 86.8% and specificity was 88.4%. The NICE FIT authors additionally proposed using f-Hb thresholds at the limit of detection (2 μg/g) for HM-JACKarc, whilst other have also suggested using the limit of quantification (7 μg/g) [12]. Such strategies are associated with fewer false negative results but with a concomitant need for more invasive investigations. Furthermore, there have been concerns that the imprecision of f-Hb at such concentrations with current techniques may lead to spurious results [21].

Whilst FIT alone has been shown to be a very accurate predictor of colorectal cancer in symptomatic patients, there is a small rate of false negative results which could lead to missed cancer diagnoses. Prior studies have attempted to combine f-Hb with other risk factors including age and sex to improve the diagnostic utility of FIT, with mixed results [22]. In the study by McSorley et al. [4] which included 4841 symptomatic patients from three Scottish health boards who underwent colonoscopy and had a FIT submitted from primary care, 14 (0.6%) patients with a normal FIT (< 10 µg/g) were diagnosed with a colorectal malignancy. Nine of these 14 patients (64.3%) were anaemic at the time of referral, and it was suggested that anaemia may be helpful in reducing the false negative rate of FIT for colorectal cancer detection in symptomatic patients.

In the present study, we found that using both FIT with a f-Hb threshold of 10 µg/g and the presence or absence of anaemia increased sensitivity for colorectal cancer from 91.80% to 98.28% and NPV from 99.87% to 99.96%. The corresponding specificity and PPV decreased from 80.42% to 65.44% and 5.47% to 3.99% respectively, while the number needed to scope to diagnose one colorectal cancer increased from 18 to 26, which we feel is acceptable. Other studies have considered combining FIT and anaemia including that by Chapman et al. [24]. Of 1106 patients referred on an urgent 2-week suspected cancer pathway with accompanying FIT, a f-Hb threshold of > 4 µg/g gave a sensitivity and specificity for colorectal cancer of 97.5% and 64.5% respectively. By combining FIT > 4 µg/g and/or the presence of anaemia, sensitivity rose to 100% and specificity dropped to 45.3%. However, patients with rectal bleeding and those referred out with a 2-week wait pathway were excluded. Bailey et al. [23] reported on 13,361 FIT studies submitted from primary care as part of their suspected colorectal cancer referral pathway. Patients with f-Hb ≥ 10 µg/g met the threshold for urgent 2-week wait investigation. Of note, those with a f-Hb greater than 4 µg/g but less than 10 µg/g in the presence of anaemia, low ferritin or thrombocytosis were also eligible for urgent investigation. Ten patients (CRC rate 0.6%) with a f-Hb 4–9.9 µg/g were ultimately diagnosed with a colorectal cancer. Five of these 10 patients were anaemic, and 6 had a low ferritin with 0 patients therefore not meeting urgent investigation criteria.

Anaemia in isolation, and in particular iron deficiency anaemia, is well recognised to be associated with colorectal cancer and would usually prompt urgent referral [25]. The overall rate of IDA in this cohort was relatively low at 5.4% for several reasons. Firstly, we have only included symptomatic patients, so no cases of asymptomatic IDA are represented. Secondly, we have chosen to present IDA as an objective parameter based on blood results, rather than as a reason for referral. It is very common to find that patients referred with “IDA” in fact have normocytic anaemia and a normal ferritin. Finally, we have used a strict definition for IDA at ferritin < 15 µg/L. There is wide variability in how iron deficiency is defined. NICE recommend a ferritin of < 30 µg/L to confirm the diagnosis of IDA but do concede that the interpretation of a ferritin can be difficult as it may be raised in the presence of inflammation [26]. Hamilton et al. [27], who refined the risk of colorectal cancer associated with anaemia, used a ferritin < 20 µg/L. The British Society of Gastroenterology guidelines state that a “serum ferritin < 15 µg/L is highly specific for iron deficiency (specificity 0.99),” and we have followed this threshold. The existing evidence and the results of the present study suggest that haemoglobin, without ferritin or other measures of iron status, could provide additional sensitivity to f-Hb for the detection of colorectal cancer in symptomatic patients. Of note, most patients with cancer who were anaemic had normocytic anaemia which has previously been established [28]. Additionally, when FIT at a f-Hb threshold of 10 µg/g was combined with IDA (ferritin < 15) in a similar manner to our combined FIT and anaemia measure, a less significant improvement in sensitivity was achieved (94.83%) (Supplementary Table 3). We therefore propose combining f-Hb with all anaemia as simpler and superior measure.

This study has a number of strengths. We present real-life practice in GG&C health board following introduction of FIT as a tool to guide referral to colorectal and gastroenterology services. We report not only on patients with FIT samples submitted as part of a referral but also on non-referred patients. We have used this to establish that FIT is actively influencing referral and investigation decisions. While other studies have included FIT from referred and non-referred patients [12, 15, 23], a particular strength of the current study is the longer median follow up of 23 months, with linkage to cancer registry data to minimise the likelihood of missed cases. Additionally, the inclusion of patients with high- and low-risk symptoms and with and without rectal bleeding reflects the most up-to-date evidence and real-life use of FIT. We have combined FIT and anaemia to form a highly effective way of excluding colorectal cancer. There are however limitations. The retrospective nature of the study meant that information on patient symptoms and co-morbidities were only available if the patient was referred to the colorectal or gastroenterology service as this information was obtained from referral letters. Although cancer registry linkage is robust, it is possible that cases of cancer in those not further investigated were missed. Finally, the nature of the study meant that other significant bowel disease including advanced adenoma and inflammatory bowel disease were not included.

## Conclusion

In NHS GG&C, GP referral pattern and secondary care investigation patterns were influenced by FIT. The addition of a normal haemoglobin concentration from a full blood count to a f-Hb < 10 μg/g was able to effectively exclude colorectal cancer in 99.96% of cases, providing excellent reassurance to GP’s and to specialist practitioners who must prioritise access to endoscopy services, particularly in the context of the current COVID pandemic and recovery period. Patients with a f-Hb < 10 μg/g and without anaemia represented 64.5% of patients. With appropriate safety netting in place, these patients can be reassured.

## Supplementary Information

Below is the link to the electronic supplementary material.

Supplementary file1 (DOCX 25 kb)

## Data Availability

Sharing of anonymised may be available upon request and subject to additional permissions.
