# Supplementary material for: Combining the quantitative faecal immunochemical test and full blood count reliably rules out colorectal cancer in a symptomatic patient referral pathway
Source: Int J Colorectal Dis. 2021 Dec 21;37(2):457–66. doi: 10.1007/s00384-021-04079-2 (PMC8803704; doi:10.1007/s00384-021-04079-2)
Supplement: Supplementary file 1 — Supplementary file1 (DOCX 25 kb) [file 384_2021_4079_MOESM1_ESM.docx]

**Supplementary Data**

Supplementary Table 1. Comparison between the Not Referred and Referred groups.

|  | | Not Referred | Referred | P |
| --- | --- | --- | --- | --- |
| N | | 2534 | 2434 |  |
| Age | Median (range) | 57 (16-97) | 60 (16-95) | <0.001 |
|  | <50 | 841 (33.2%) | 614 (25.2%) |  |
|  | 50-74 | 1284 (50.7%) | 1368 (56.2%) |  |
|  | ≥75 | 409 (16.1%) | 542 (18.6%) |  |
| Sex | Male | 1042 (41.1%) | 1060 (43.5%) | 0.083 |
|  | Female | 1492 (58.9%) | 1374 (56.5%) |  |
| SIMD | 1 (most deprived) | 691 (27.3%) | 795 (32.7%) | <0.001 |
|  | 2 | 434 (17.1%) | 434 (17.8%) |  |
|  | 3 | 329 (13.0%) | 292 (12.0%) |  |
|  | 4 | 399 (15.7%) | 367 (15.1%) |  |
|  | 5 (least deprived) | 681 (26.9%) | 546 (22.4%) |  |
| Medications | Aspirin | 397 (15.7%) | 539 (22.1%) | <0.001 |
|  | NSAIDs | 267 (10.5%) | 301 (12.4%) | 0.043 |
|  | ACE Inhibitors | 382 (15.1%) | 470 (19.3%) | <0.001 |
|  | Statins | 562 (22.2%) | 688 (28.3%) | <0.001 |
|  | H2 Antagonists | 72 (2.8%) | 87 (3.6%) | 0.142 |
|  | Metformin | 72 (2.8%) | 87 (3.6%) | 0.142 |
|  | Oral Anticoagulants | 73 (2.9%) | 122 (5.0%) | <0.001 |
|  | Anti-spasmodics | 685 (27.0%) | 642 (26.4%) | 0.601 |
| f-Hb (µg/g) | <10 | 2355 (92.9%) | 1413 (58.1%) | <0.001 |
|  | 10-149 | 32 (1.3%) | 603 (24.8%) |  |
|  | 150-399 | 5 (0.2%) | 108 (4.4%) |  |
|  | ≥400 | 10 (0.4%) | 211 (8.7%) |  |
|  | N/A | 132 (5.2%) | 99 (4.1%) |  |
| Anaemia* | No | 1708 (82.4%) | 1676 (77.6%) | <0.001 |
|  | Yes | 365 (17.6%) | 483 (22.4%) |  |
| Iron Deficiency Anaemia (Ferritin <15)# | No | 1962 (96.1%) | 1988 (93.2%) | <0.001 |
|  | Yes | 80 (3.9%) | 146 (6.8%) |  |
| Anaemia and MCV^£^ | Not Anaemic | 1708 (82.4%) | 1676 (77.6%) | <0.001 |
|  | Macrocytic Anaemia (MCV >100) | 28 (1.4%) | 27 (1.3%) |  |
|  | Normocytic Anaemia  (MCV 80-100) | 295 (14.2%) | 370 (17.1%) |  |
|  | Microcytic Anaemia  (MCV <80) | 42 (2.0%) | 86 (4.0%) |  |
| Colorectal Cancer | | 1 (0.04%) | 60 (2.5%) | <0.001 |

*Data missing for 736 (14.8%) patients.

# Data missing for 792 (15.9%) patients.

£ Data missing for 736 (14.8%) patients.

Supplementary Table 2. Comparison between Referred but not Scoped and Referred and Scoped groups.

|  | | Referred but not Scoped | Referred and Scoped | P |
| --- | --- | --- | --- | --- |
| N | | 1104 | 1330 |  |
| Age | Median (range) | 61 (16-95) | 60 (17-94) | <0.001 |
|  | <50 | 281 (25.5%) | 333 (25.0%) |  |
|  | 50-74 | 578 (52.4%) | 790 (59.4%) |  |
|  | ≥75 | 245 (22.2%) | 207 (15.6%) |  |
| Sex | Male | 484 (43.8%) | 576 (43.3%) | 0.792 |
|  | Female | 620 (56.2%) | 754 (56.7%) |  |
| SIMD | 1 (most deprived) | 349 (31.6%) | 446 (33.5%) | 0.274 |
|  | 2 | 197 (17.8%) | 237 (17.8%) |  |
|  | 3 | 120 (10.9%) | 172 (12.9%) |  |
|  | 4 | 175 (15.9%) | 192 (14.4%) |  |
|  | 5 (least deprived) | 263 (23.8%) | 283 (21.3%) |  |
| Co-morbidity* | Respiratory Disease | 112 (17.5%) | 165 (16.1%) | 0.479 |
|  | Diabetes | 80 (12.5%) | 122 (11.9%) | 0.741 |
|  | Cardiovascular Disease | 106 (16.5%) | 153 (15.0%) | 0.391 |
|  | IBD | 4 (0.6%) | 2 (0.2%) | 0.156 |
| Medication | Aspirin | 259 (23.5%) | 280 (21.1%) | 0.154 |
|  | NSAIDs | 128 (11.6%) | 173 (13.0%) | 0.292 |
|  | ACE Inhibitors | 202 (18.3%) | 268 (20.2%) | 0.249 |
|  | Statins | 310 (28.1%) | 378 (28.4%) | 0.852 |
|  | H2 Antagonists | 48 (4.3%) | 39 (2.9%) | 0.061 |
|  | Metformin | 48 (4.3%) | 39 (2.9%) | 0.061 |
|  | Oral Anticoagulants | 63 (5.7%) | 59 (4.4%) | 0.153 |
|  | Anti-spasmodics | 317 (28.7%) | 325 (24.4%) | 0.017 |
| Symptoms | Any Red Flag | 993 (89.9%) | 1201 (90.3%) | 0.77 |
|  | Rectal Bleeding | 301 (27.3%) | 586 (44.1%) | <0.001 |
|  | Persistent Diarrhoea | 237 (21.5%) | 365 (27.4%) | 0.001 |
|  | Other Change in Bowel Habit | 567 (51.4%) | 640 (48.1%) | 0.112 |
|  | Weight Loss | 237 (21.5%) | 229 (17.2%) | 0.008 |
|  | Abdominal Pain | 395 (35.8%) | 401 (30.2%) | 0.003 |
|  | Anal Pain | 44 (4.0%) | 33 (2.5%) | 0.035 |
|  | Faecal Soiling | 65 (5.9%) | 85 (6.4%) | 0.607 |
|  | Rectal Mass | 18 (1.6%) | 26 (2.0%) | 0.55 |
|  | Abdominal Mass | 24 (2.2%) | 36 (2.7%) | 0.399 |
| FIT | <10 | 841 (76.2%) | 572 (43.0%) | <0.001 |
|  | 10-149 | 170 (15.4%) | 433 (32.6%) |  |
|  | 150-399 | 19 (1.7%) | 89 (6.7%) |  |
|  | ≥400 | 30 (2.7%) | 181 (13.6%) |  |
|  | N/A | 44 (4.0%) | 55 (4.1%) |  |
| Anaemia# | No | 750 (77.1%) | 926 (78.1%) | 0.58 |
|  | Yes | 223 (22.9%) | 260 (21.9%) |  |
| Iron Deficiency Anaemia (Ferritin <15)^¶^ | No | 909 (94.6%) | 1079 (92.0%) | 0.018 |
|  | Yes | 52 (5.4%) | 904 (8.0%) |  |
| Anaemia and MCV^£^ | Not Anaemic | 750 (76.9%) | 926 (78.2%) | 0.088 |
|  | Macrocytic Anaemia (MCV >100) | 30 (3.1%) | 56 (4.7%) |  |
|  | Normocytic Anaemia  (MCV 80-100) | 183 (18.8%) | 187 (15.8%) |  |
|  | Microcytic Anaemia  (MCV <80) | 12 (1.2%) | 15 (1.3%) |  |
| Colorectal Cancer | | 4 (0.4%) | 56 (4.2%) | <0.001 |

*Data missing for 771 (31.7%) patients.

#Data missing for 275 (11.3%) patients.

¶Data missing for 300 (12.3%) patients.

£Data missing for 275 (11.3%) patients.

Supplementary Table 3. Comparison by combined FIT and iron deficiency anaemia with a valid FIT, full blood count and ferritin.

|  | | f-HB <10µg/g  No IDA | f-Hb <10µg/g  IDA | f-Hb≥10µg/g  No IDA | f-Hb≥10µg/g  IDA | P |
| --- | --- | --- | --- | --- | --- | --- |
| N | | 2987 | 152 | 793 | 64 |  |
| Age | Median (range) | 59 (16-94) | 56 (23-91) | 63 (17-97) | 64 (19-95) | <0.001 |
|  | <50 | 878 (29.5%) | 44 (29.3%) | 189 (23.9%) | 16 (25.0%) |  |
|  | 50-74 | 1648 (55.4%) | 82 (54.7%) | 395 (50.0%) | 27 (42.2%) |  |
|  | ≥75 | 450 (15.1%) | 24 (16.0%) | 206 (26.1%) | 21 (32.8%) |  |
| Sex | Male | 1247 (41.9%) | 38 (25.3%) | 365 (46.2%) | 21 (32.8%) | <0.001 |
|  | Female | 1729 (58.1%) | 112 (74.7%) | 425 (53.8%) | 43 (67.2%) |  |
| Colorectal Cancer | | 3 (0.1%) | 2 (1.3%) | 40 (5.0%) | 13 (20.3%) | <0.001 |
